# Comprehensive Evaluation of the Antibacterial and Antifungal Activities of *Carlina acaulis* L. Essential Oil and Its Nanoemulsion

**DOI:** 10.3390/antibiotics10121451

**Published:** 2021-11-25

**Authors:** Antonio Rosato, Alexia Barbarossa, Ahmed M. Mustafa, Giulia Bonacucina, Diego Romano Perinelli, Riccardo Petrelli, Filippo Maggi, Eleonora Spinozzi

**Affiliations:** 1 Department of Pharmacy-Drug Sciences, University of Bari “Aldo Moro”, 70126 Bari, Italy; antonio.rosato@uniba.it (A.R.); alexia.barbarossa@uniba.it (A.B.); 2 Department of Pharmacognosy, Faculty of Pharmacy, Zagazig University, Zagazig 44519, Egypt; ahmed.mustafa@unicam.it; 3 School of Pharmacy, University of Camerino, via Sant’ Agostino 1, 62032 Camerino, Italy; giulia.bonacucina@unicam.it (G.B.); diego.perinelli@unicam.it (D.R.P.); riccardo.petrelli@unicam.it (R.P.); eleonora.spinozzi@unicam.it (E.S.)

**Keywords:** *Carlina acaulis*, carlina oxide, formulation, MIC, clinical isolates

## Abstract

Plants are considered to be an excellent source of new compounds with antibiotic activity. *Carlina acaulis* L. is a medicinal plant whose essential oil (EO) is mainly characterized by the polyacetylene carlina oxide, which has antimicrobial properties. The aim of this study was to evaluate the antimicrobial and antifungal activities of *C. acaulis* EO, carlina oxide, and nanoemulsion (NE) containing the EO. The EO was obtained through plant roots hydrodistillation, and carlina oxide was purified from it through silica gel column chromatography. The NE containing *C. acaulis* EO was prepared with the high-pressure homogenization method, and the minimum inhibitory concentration (MIC) was determined against several bacterial and fungal strains for all the *C. acaulis*-derived products. The latter resulted active versus all the screened Gram-positive bacterial strains and also on all the fungal strains with low MIC values. For yeast, the EO and carlina oxide showed good MIC values. The EO-NE demonstrated a better activity than the pure EO on all the tested bacterial and fungal strains. The results suggest that *C. acaulis*-derived products could be potential candidates for the development of natural antibacterial and antifungal agents.

## 1. Introduction

One of the major concerns of today’s world is the increasing antibiotic resistance that is threatening our ability to combat infectious diseases, and, for this reason, new antibiotic agents are urgently needed. In this scenario, plants represent a good source of bioactive compounds due to the production of secondary metabolites that act as defensive molecules against microbes [1]. In fact, several antibiotic agents have been obtained from different plant species, and the majority of the antibiotics available are represented by compounds of a natural origin [2,3].

*Carlina acaulis* L., belonging to the Asteraceae family, is a plant with proven biological importance. It is native to the dry and calcareous soils of the mountains of southern and central Europe (from 700 to 2000 m of altitude). *C. acaulis* is a monocarpic perennial herb and is also called ‘carciofo selvatico’ (wild artichoke) or ‘piccolo cardo’ (little thistle, stemless carline thistle) [4]. From the roots of this plant, an essential oil (EO) can be obtained whose distinctive feature is the presence of the polyacetylene carlina oxide as the main constituent (> 95%).

A significant traditional medicinal application of this plant is the cure of dermatological conditions, such as skin exudates and pustules [5], scars and cracked skin [6], and sunburn, in combination with sulfur [7]. In addition, it is also used for the treatment of ulcerations, skin infections, and swellings [8,9].

Moreover, several studies report the activity of this plant against different microorganisms. For instance, Kernóczi et al. [10] reported a moderate activity against some Gram-positive bacteria, while Belabbes et al. [11] reported an antifungal activity of the EO against *Penicillium expansum* and *Aspergillus niger*. The antimicrobial activity of the EO and decoction obtained from the roots was also significant against *Staphylococcus aureus* [12]. Extracts of *C. acaulis* roots were reported as particularly active on *Klebsiella pneumoniae* and *Escherichia coli* and on *Candida albicans* [13]. Moreover, carlina oxide was also found highly effective against *Streptococcus pyogenes*, *Pseudomonas aeruginosa*, *C. albicans*, and *C. glabrata* [14].

Both the EO and carlina oxide are also reported for their various biological activities, such as antitripanosomal [14], cytotoxic [15], and insecticidal effects [16,17,18]. Besides the biological properties mentioned above, *C. acaulis* has also a long history in traditional medicine as an anthelmintic and diuretic, antiulcer, diaphoretic, laxative, and emetic agent [5]. Despite the strong and promising activity of carlina oxide, its mode of action is still unexplored. However, members of the chemical class of polyacetylenes represent, for the family of Asteraceae, important defensive agents. In detail, the presence of conjugate double bonds and a triple bond in carlina oxide could be responsible for the production of reactive oxygen species (ROS) and phototoxicity, also known for other aromatic polyacetylenes [19]. These compounds are highly reactive due to their oxidation and degradation, which depend on the exposure to UV light but also on the pH [20]. The high lipophilicity of carlina oxide could also allow it to enter into the cells and interact with amino groups of biomolecules.

Considering the biocidal potential reported for *C. acaulis* in ethnopharmacology and some preliminary studies, the aim of this work was to evaluate the antibacterial and antifungal activities of its EO and carlina oxide against different bacteria and yeasts. Moreover, in order to improve the physicochemical properties of the EO and its chemical stability, an EO-nanoemulsion (NE) was developed and tested as well for the first time.

## 2. Results

### 2.1. Chemical Analysis of C. acaulis Essential Oil

**Figure 1:**
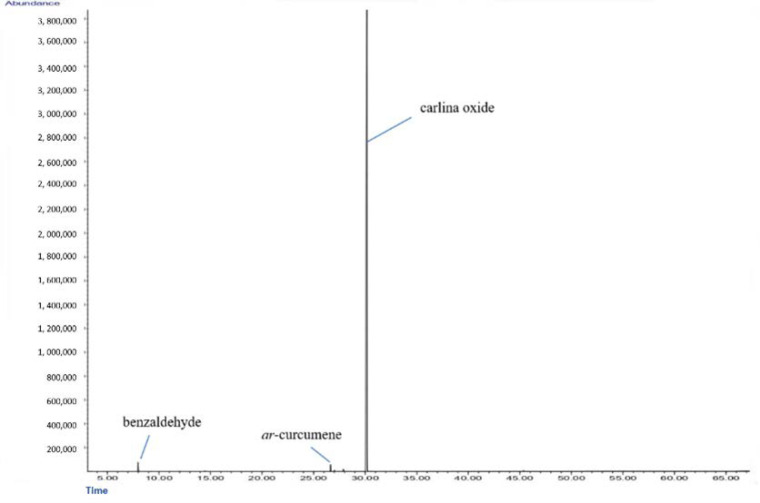
Gas chromatography–mass spectrometry (GC–MS) chromatogram of *C. acaulis* essential oil.

The chemical analysis conducted on *C. acaulis* EO led to a composition dominated by the presence of carlina oxide (97.8%) (Figure 1). Other minor constituents were benzaldehyde (0.9%), *ar*-curcumene (0.7%), *β*-sesquiphellandrene (0.2%), and *α*-zingiberene (0.1%). The total identified compounds accounted for 99.8%. The chemical profile of the EO was linear with those reported in the literature [17,21].

### 2.2. Nanoemulsion Development

After a preliminary evaluation, NE composed of 6% *w/w* of *C. acaulis* EO, 2% of ethyl oleate, and 3% *w/w* of polysorbate 80 was chosen to be tested for its antibacterial and antifungal activity. The present NE was optimized starting from the nanoemulsifed system based on *C. acaulis* EO as reported in Benelli et al. [16]. The addition of 2% of ethyl oleate allowed the reduction of the polysorbate 80 concentration from 4 to 3% *w/w*, maintaining the average size of the internal droplets of the NE below 200 nm (Z-average from DLS in the range 114–136 nm) and the absence of oil droplets above 1 µm (observed from optical microscope) as in the previous work [16]. The physical stability of the NE was also retained, at least up to 9 months, without showing phase separation and creaming. No increase in the mean particle size (Z-average) and in the polydispersity index (PDI) occurred, as indicated by the DLS measurements (Table 1).

### 2.3. Antimicrobial Assay

The antimicrobial assays revealed an activity of *C. acaulis* EO and carlina oxide versus the screened Gram-positive bacterial strains and also on the fungal strains.

In detail, the EO and carlina oxide were active against the Gram-positive bacteria with values ranging between 2.7 and 10.9 mg/mL and 0.33 and 5.35 mg/mL. Interestingly, a significant activity was detected on clinical bacterial isolates with remarkable characteristics of virulence, such as on all the *Staphylococcus* spp. strains (23, 24, IAC, TER, *sciuri*, *lugdunensis*, *warneri*) (Table 2), carlina oxide being more effective than the EO.

Conversely, the tests showed a complete EO and carlina oxide inefficacy against the Gram-negative species. Regarding the EO-NE, it was effective on the bacterial Gram-positive strains, with MIC values ranging between 7.5 and 60 mg/mL, while the effect against the Gram-negative strains resulted in lower values, with the MIC values ranging between 125 and 500 mg/mL.

On the other hand, a strong and homogenous action of the EO and carlina oxide was obtained against the fungal strains considered in this study, both being capable of inhibiting the growth of all the *Candida* spp. isolates. The EO showed a mild activity only against some fungal strains, while the carlina oxide showed a strong fungicidal activity (Table 3). In detail, for yeasts, the EO and carlina oxide MICs ranged between 0.34 and 2.7 mg/mL, and 0.02 and 0.08 mg/mL, respectively (Table 3).

The best efficacy for the carlina oxide was detected also against clinically isolated yeasts, such as the *Candida* species.

Regarding the EO-NE, it was effective on yeasts with MIC values ranging between 0.9 and 1.9 mg/mL.

The agar diffusion method performed according to the CLSI protocol [22] to assess the activity of the EO confirmed the results obtained by the microdilution method. The results are illustrated in Table 3 and correlate perfectly with the MIC values.

## 3. Discussion

*C. acaulis* is widely used in traditional medicine, also being cited by several pharmacopoeias [23,24] for its medicinal properties against several diseases. This plant is also enclosed in the Italian list of botanicals to be used in food supplements [25] and in the BELFRIT (Belgium France Italy) list [26]. Some studies on the antibacterial and antifungal properties of this plant have been reported, showing promising activities not only against *K. pneumoniae* and *E. coli* [13], but also against *S. pyogenes* and *P. aeruginosa* [14]. This plant was also reported to be effective against some fungal strains, such as *P. expansum* and *A. niger* [11], *C. albicans*, and *C. glabrata* [13,14].

Even though some antibacterial and antifungal activities of *C. acaulis* are reported in the literature, this study provides deep insight regarding the antibacterial and antifungal activity of its EO and its main constituent, carlina oxide. Moreover, the *C. acaulis* EO encapsulated into an NE has been tested for the first time as an antibacterial and antifungal agent. Our results showed that the EO and carlina oxide are active against Gram-positive bacteria, with a more marked activity for carlina oxide. This difference in activity has also been detected on some clinical bacterial isolates with remarkable characteristics of virulence, against which carlina oxide was more effective. Concerning the activity on fungal strains, both products were active, with a stronger activity for carlina oxide. The obtained results are in a good agreement with the literature; for example, Stojanović-Radić et al. [12] reported that both the EO and decocts of *C. acanthifolia* L. exhibited a very high antimicrobial activity against all the tested microbial strains, with *S. aureus* being the most sensitive one. In addition, Herrmann et al. (2011) confirmed the strong activity of carlina oxide of *C. acaulis* against *S. pyogenes*, *P. aeruginosa*, *C. albicans*, and *C. glabrata* [14].

Our study demonstrated also that the encapsulation of the EO into an NE leads to an improvement of its antibacterial and antifungal properties. In the case of Gram-positive bacteria, the MIC values of the EO-NE resulted in higher values than the one of the EO (between 7.5 and 60 and 0.68 and 2.9 mg/mL, respectively), but, considering that only the 6% (*w/w*) of the EO was encapsulated, it resulted in actually more activity than the pure EO. Moreover, the activity of the EO, which was completely absent against the Gram-negative bacteria, was detected for its encapsulated form, which showed MIC values between 125 and 500 mg/mL. Finally, the EO-NE resulted also in more activity than the pure EO against the fungal strains (between 0.9 and 1.9 and 0.34 and 2.7 mg/mL, respectively). These results confirm the importance of nanoformulations for the improvement not only of the stability and physicochemical properties of natural products but also of their biological properties. The enhancement of the EO activity encapsulated in the NE could be attributed to its best dispersion in the aqueous phase, as well as improved interactions with the target. In addition, the EO droplets encapsulated into the NE have a small size and large surface area, which enhances their absorption and cellular penetration. This work demonstrates the importance of natural products as a source of new bioactive compounds and may be the starting point for the development of new antibacterial and antifungal agents based on *C. acaulis* derivatives.

## 4. Materials and Methods

### 4.1. Essential Oil Isolation and Purification of Carlina Oxide

Commercial dry roots of *C. acaulis* (lot no C-210920250920) were acquired from Minardi & Figli S.r.l. (Bagnacavallo, Ravenna, Italy; https://www.minardierbe.it/, accessed on 24 July 2021) and were collected from an Albanian accession of spontaneously growing plants harvested in 2020. Before the extraction process, the roots were shredded into a 1.5 mm size using a grinder from Albrigi (Stallavena, Verona, Italy; mod. E0585), and then they were soaked overnight into a 10 L round bottom flask filled with 7 L of distilled water. Afterwards, these roots were subjected to a hydrodistillation process for 8 h using a Clevenger-type device, which was heated using a Falc MA mantle (Falc Instruments, Treviglio, Italy). The EO yield was 0.76% (*w/w*), and it showed a yellowish color and a density of 1.063 g/mL. For the isolation of carlina oxide, 1.403 g of EO were purified by silica gel (70 g) column chromatography (70−230 mesh, 60 Å, Merck) with 100% of *n*-hexane (Merk, Italy) as mobile phase to obtain 1.306 g of pure carlina oxide. Its structure was confirmed through mass spectrometry (MS) and NMR analyses, and the results were comparable with those reported in literature [21].

### 4.2. Chemical Characterization of the Essential Oil

*C. acaulis* essential oil was analyzed using an Agilent 8890 gas chromatograph (GC) equipped with a single quadrupole 5977B mass spectrometer (Santa Clara, California, USA) and an autosampler PAL RTC120 (CTC Analytics AG, Zwingen, Switzerland). The ionization was obtained by using an electron ionization source (EI). The injector temperature was set at 280 °C, and the gas carrier was helium at flow rate of 1 mL min^−1^.

The separation of the molecules was achieved using an HP-5 capillary column (30 m, 250 µm i.d., 0.25 µm film thickness), which was thermostated at 60 °C for 5 min, then raised up to 220 °C at 4 °C/min, then to 280 °C at 11 °C/min and held for 15 min, and finally to 300 °C at 15 °C/min and held for 0.5 min. The run time was about 67 min. The transfer line was set at 280 °C, and the temperatures of the ionization source and the mass analyzer were set at 230 and 150 °C, respectively. The acquisition was carried out in SCAN mode (29–400 m/z). The EO was diluted (1:250) in *n*-hexane (Merk, Italy) and 1 µL injected in split mode (1:50). The chromatogram was analyzed using the MSD ChemStation software (Agilent, Santa Clara, CA, USA, Version G1701DA D.01.00), and the NIST Mass Spectral Search Program (NIST, Gaithersburg, MD, USA) for the NIST/EPA/NIH EI and NIST Tandem Mass Spectral Library v. 2.3 (NIST, Gaithersburg, MD, USA) were used to analyze data. The identification of the EO components was achieved by the combination of the temperature-programmed retention indices (RIs) and mass spectra (MS) compared with those of ADAMS, NIST 17, and FFNSC2 libraries [27,28,29]. RI was calculated using a mix of *n*-alkanes (C_8_-C_30_, Supelco, Bellefonte, CA, USA) according to the Van den Dool and Kratz [30] formula.

### 4.3. Nanoemulsion Preparation and Characterization

The NE containing 6% *w/w* of *C. acaulis* EO, 2% of ethyl oleate, and 3% *w/w* of Polysorbate 80 (Merk, Italy) was prepared according to the high-pressure homogenization method (French pressure cell press; American Instrument Company, AMINCO, Silver Spring, MD, USA) and characterized using dynamic light scattering (DLS; Malvern Instrument, Malvern, UK) and optical microscope (Invenio 3S, DeltaPix, Hassellunden, Denmark) as previously reported [16].

### 4.4. Antimicrobial Assay

The antibacterial and antifungal activity of *C. acaulis* EO, carlina oxide, and NE were tested against various bacterial and fungal strains, with particular attention to Gram-positive and Gram-negative bacteria, together with yeast. The isolation and identification procedures were executed by the Department of Biomedical Science and Human Oncology, Hygiene Section, University of Bari, Italy. All the isolates derived from patients admitted to the intensive care unit of Department of Biomedical Science and Human Oncology. The bacterial and fungal strains were identified using conventional physiological and morphological methods (API systems). The ATCC strains were: *Bacillus cereus* ATCC 10876, *Staphylococcus aureus* ATCC 29213, ATCC 6538, ATCC 6538P, ATCC 25923 and ATCC 43300 methicillin resistant (MRSA), *Enterococcus faecalis* ATCC 29212, *Escherichia coli* ATCC 25922, 35218, *Acinetobacter baumannii* ATCC 19606, *Pseudomonas aeruginosa* ATCC 27853, *Escherichia coli* ATCC 25922, and *Klebsiella pneumoniae* ATCC 13883.

An additional set of bacteria derived from clinical isolation was represented by: *Corynebacterium striatum* RM, *Proteus mirabilis* IG, *Cytrobacter freundii* IG, *Enterococcus faecalis* BS, 2011, 1011, *Staphylococcus aureus* IG24, IG23, IAC, TER, *Lugdunensis sciuri*, and *Serratia marcescens* IG. Moreover, five clinically isolated strains with serious profile of multi-resistance to antibiotics were also taken into consideration: *Acinetobacter bambini* BS, *Escherichia coli* ESBL, and *Klebsiella pneumoniae* BS. On the other hand, the yeast strains considered for this study were: strains from the ATCC, such as *C. albicans* ATCC 10231, *C. albicans* ATCC 90028, *C. glabrata* ATCC 15126, *C. tropicalis* ATCC 750, *C. kefyr* ATCC 204093, and *C. krusei* ATCC 6258. The fungal strains from clinical isolation were identified as *C. albicans* A18, 10A12,810, *C. krusei* 31A29, *C. parapsilosis* 11A13, 1A1, 911, 910, and *C. tropicalis* 810.

In this study, modified minimum inhibitory concentration (MIC) determinations and agar diffusion procedure were used according to the Clinical Laboratory Standards Institute (CLSI, 2015, 2008) guidelines. MIC values were reported in mg/mL and were compared to MIC values of the standard antibacterial drug Rifaximin (μg/mL) and of the standard antifungal drug Amphotericin (μg/mL) [31].

The EO and carlina oxide two-fold serial dilutions were plated for bacteria in the suitable test medium ranged between 0.17 and 10.9, 0.17 and 10.7 mg/mL, respectively. For tests on yeast, different dilutions were prepared: 0.17–5.4 mg/mL for the EO and 0.02–0.16 mg/mL for carlina oxide. The NE ranged between 0.5 mg and 500 mg for bacteria and yeast. To be sure that the solvent had no adverse effect on bacterial and yeast growth, a control test was carried out by using ethanol (Merk, Milan, Italy) (5%) at its maximum concentration along with the medium. The NE was used as it was without the use of solvent.

The bacterial species were cultured on Mueller Hinton Agar (MHA, Oxoid), and each bacterial suspension was composed of 2–3 colonies of each strain taken from an MHA plate and dissolved in 2 mL of Mueller Hinton Broth (MHB, Oxoid). The MICs of *C. acaulis* EO and of carlina oxide were determined by the broth microdilution method, using 96-well plates, according to CLSI guidelines [31] for bacteria with some modifications.

The turbidity of bacterial cell suspension was analyzed at a wavelength of 625 nm using spectrophotometric method (Thermo Spectro Nic, Genesis 20), while it should be 0.08 to 0.10 for the 0.5 McFarland standard, corresponding approximately to 108 colony-forming units/mL (CFU/mL). Then, the standardized suspension was diluted 1:100 with MHB to have 1–2 × 10^6^ CFU/mL. All wells were seeded with 100 µL of inoculum. In addition, few wells containing only inoculated broth as control growth were prepared. The plates were incubated at 37 °C for 24 h, and the MIC values were recorded as the last well containing no bacterial growth. The MIC was determined by using an antibacterial assay repeated in duplicate. The MHB medium well control 0.1% (*v/v*) Tween 80 and ethanol (Merk, Milan, Italy) 3% (without the EO) was used as positive growth control. MIC was defined as the lowest concentration that did not result in any visible growth of the bacterial strains compared to their growth in the control well [32].

The fungal species were sub-cultured twice on Sabouraud dextrose agar before being tested. Each fungal suspension was taken from its frozen stock at –70 °C and yeast cells were washed four times in sterile saline. The strains were inoculated in tubes containing 5 mL of Sabouraud dextrose broth and then incubated under stirring at 35 °C for 48 h. MIC values were determined by broth microdilution method in accordance with CLSI guidelines [31] for yeasts. For the preparation of the EO for the antifungal tests, the same procedure used for the antibacterial tests was followed. A small amount of inoculum from incubated tubes was dissolved in RPMI 2% glucose and then spectrophotometrically adjusted to 0.5 × 103 to 2.5 × 10^3^ CFU/mL (McFarland, turbidity standard). The initial inoculum was confirmed by plating serial dilutions and determining the colony counts. A total of 0.1 mL of each yeast suspension was dispensed into serially diluted wells containing the drugs or the EO, achieving final drug concentration. After the addition of 0.1 mL of inoculum, the plates were incubated at 36 °C for 48 h. MIC was defined as the lowest concentration of the mixtures at which no visible growth of the fungal strains could be detected compared to their growth in the negative control well. MIC values were reported in mg/mL for the EO, NE, and carlina oxide and in μg/mL for antimicrobial drugs, respectively. The results of Agar diffusion method were reported as mm of the inhibition zone. MIC and Agar diffusion determinations were realized in triplicate in three independent assays. The results are averages of three evaluations on three different days, reported in Table 2 and Table 3.

### 4.5. Statistical Analyses

Analyses were performed in triplicate, and the results were expressed as mean ± standard deviation (SD) using Microsoft Excel (Microsoft Office 2007, Redmond, WA, USA).

## 5. Conclusions

The misuse and overuse of antibiotics in humans and animals against pathogens has led to the occurrence of resistant and multidrug-resistant microorganisms [33]. Therefore, there is the urgent need for alternatives to the commonly used antibiotics and, nowadays, the interest of research is increasingly focusing on botanical products, particularly EOs. These have shown several biological properties and antimicrobial activities, resulting in effectiveness also against multidrug-resistant strains [34]. The antimicrobial actions of EOs are mostly due to their chemical composition, and the combination of these different compounds usually leads to a reduction in the emergence of resistance. The use of these botanical products is characterized by sustainability and low toxicity, but it is also accompanied by limitations due to their physicochemical properties [35]. For this reason, encapsulation systems are usually adopted not only to improve the limits presented by EOs but also to improve their biological activity. In our study, promising antibacterial and antifungal activities were detected for *C. acaulis* EO and for its main constituent, carlina oxide, which showed the best activity against clinically isolated strains as well. This well supports the ethnopharmacological uses of the plants against skin infections and other disorders caused by microbial pathogens. Moreover, the NE, encapsulating only 6% of the EO, showed a higher activity than the pure EO, demonstrating that, often, the formulation system can improve the biological activity of the encapsulated product as well. This study lays the foundation for the development of antibacterial and antifungal agents based on *C. acaulis* derivatives.

## Figures and Tables

**Table 1:** Variation of Z-Average (nm) and polydispersity index (PDI) for the optimized nanoemulsion over time (T0-T270 days).

Time (Days)	Z-Average (nm)	PDI
T0	134.62 ± 2.22	0.268 ± 0.009
T30	129.63 ± 1.11	0.265 ± 0.005
T90	121.5 ± 0.78	0.260 ± 0.008
T180	117.63 ± 2.23	0.256 ± 0.009
T270	114.7 ± 0.69	0.262 ± 0.002

**Table 2:** Minimal inhibitory concentration (MIC) and agar diffusion (ADIFF) results of *C. acaulis* essential oil, nanoemulsion, and carlina oxide against different bacterial species. Rifaximin MIC values are expressed as µg/mL.

	EO MIC ^1^ (mg/mL)	EO ADIFF ^2^ (mm)	NE MIC ^3^ (mg/mL)	Carlina Oxide MIC ^4^ (mg/mL)	Rifaximin MIC ^5^ (μg/mL)
*Bacillus cereus 10876*	5.4	0.9	15	na ^6^	0.1
*Corynebacterium striatum RM*	5.4	1.3	15	1.33	0.4
*Enterococcus faecalis 1011*	10.9	1.0	60	5.35	0.4
*Enterococcus faecalis 29212*	5.4	0.8	60	10.7	0.1
*Staphylococcus aureus 23*	10.9	0.8	60	5.35	0.1
*Staphylococcus aureus 24*	5.4	1.0	15	0.33	0.4
*Staphylococcus aureus 25923*	2.7	0.7	15	0.33	0.1
*Staphylococcus aureus 29213*	5.4	1.1	15	0.33	0.1
*Staphylococcus aureus 43300*	2.7	0.9	7.5	1.33	3.1
*Staphylococcus aureus 6538*	2.7	1.0	7.5	1.33	0.1
*Staphylococcus aureus 6538P*	5.4	1.0	15	0.67	0.1
*Staphylococcus aureus IAC*	5.4	0.9	7.5	0.67	0.1
*Staphylococcus aureus TER*	10.9	0.7	15	1.32	0.1
*Staphylococcus lugdunensis*	10.9	1.0	15	1.33	0.1
*Staphylococcus sciuri*	10.9	1.0	15	2.77	0.1
*Staphylococcus warneri*	5.4	1.0	15	1.33	0.1
*Acinetobacter baumannii BS*	na	na	15	na	25.0
*Citrobacter freundii IG*	na	na	500	na	12.5
*Escherichia coli 25922*	na	na	125	na	6.3
*Escherichia coli 35218*	na	na	125	na	6.3
*Escherichia coli ESBL*	na	na	250	na	6.3
*Klebsiella pneumoniae 13883*	na	na	500	na	12.5
*Klebsiella pneumoniae BS*	na	na	125	na	25.0
*Proteus mirabilis*	na	na	500	na	6.3
*Pseudomonas aeruginosa 27853*	na	na	250	na	6.3
*Serratia marcescens IG*	na	na	500	na	6.3

^1^ Essential oil (EO); minimum inhibitory concentration (MIC); ^2^ essential oil (EO); agar diffusion (ADIFF); ^3^ nanoemulsion (NE); ^4^ carlina oxide minimum inhibitory concentration (MIC); ^5^ Rifaximin minimum inhibitory concentration (MIC); ^6^ na, not active.

**Table 3:** Minimal inhibitory concentration (MIC) and agar diffusion (ADIFF) results of *C. acaulis* essential oil (EO), nanoemulsion (NE), and carlina oxide against different fungal species. Amphotericin MIC values are expressed as µg/mL.

	EO MIC ^1^ (mg/mL)	EO ADIFF ^2^ (mm)	NE MIC ^3^ (mg/mL)	Carlina Oxide MIC ^4^ (mg/mL)	Amphotericin B MIC ^5^ (μg/mL)
*Candida albicans ATCC 10231*	0.68	1.30	1.9	0.04	1.0
*Candida albicans ATCC 90028*	1.35	1.00	1.9	0.04	1.0
*Candida glabrata ATCC 15126*	2.70	1.22	0.9	0.04	0.5
*Candida kefyr ATCC 200,0493*	2.70	0.91	0.9	0.02	0.5
*Candida krusei ATCC 6258*	2.70	1.10	0.9	0.02	1.0
*Candida albicans 10A12*	2.70	1.20	0.9	0.04	1.0
*Candida krusei 31A29*	0.67	1.52	1.9	0.04	1.0
*Candida parapsilosis 11A13*	0.67	1.41	1.9	0.08	0.5
*Candida parapislosis 1A1*	0.34	1.41	1.9	0.04	1.0
*Candida parapsilosis 910*	1.35	1.22	1.9	0.04	0.5
*Candida parapsilosis 911*	1.35	1.21	1.9	0.04	0.5
*Candida tropicalis 810*	0.36	0.90	1.9	0.08	1.0

^1^ Essential oil (EO); minimum inhibitory concentration (MIC); ^2^ essential oil (EO); agar diffusion (ADIFF); ^3^ nanoemulsion (NE); ^4^ carlina oxide minimum inhibitory concentration (MIC); ^5^ Amphotericin B minimum inhibitory concentration (MIC).

## Data Availability

Not applicable.
